# Gentle Masking of Low-Complexity Sequences Improves Homology Search

**DOI:** 10.1371/journal.pone.0028819

**Published:** 2011-12-19

**Authors:** Martin C. Frith

**Affiliations:** Computational Biology Research Center, Institute for Advanced Industrial Science and Technology, Koto-ku, Tokyo, Japan; National Institutes of Health, United States of America

## Abstract

Detection of sequences that are homologous, i.e. descended from a common ancestor, is a fundamental task in computational biology. This task is confounded by low-complexity tracts (such as atatatatatat), which arise frequently and independently, causing strong similarities that are not homologies. There has been much research on identifying low-complexity tracts, but little research on how to treat them during homology search. We propose to find homologies by aligning sequences with “gentle” masking of low-complexity tracts. Gentle masking means that the match score involving a masked letter is 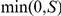, where 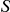 is the unmasked score. Gentle masking slightly but noticeably improves the sensitivity of homology search (compared to “harsh” masking), without harming specificity. We show examples in three useful homology search problems: detection of NUMTs (nuclear copies of mitochondrial DNA), recruitment of metagenomic DNA reads to reference genomes, and pseudogene detection. Gentle masking is currently the best way to treat low-complexity tracts during homology search.

## Introduction

The problem of false homology prediction due to low-complexity sequences is sufficiently severe that it has been addressed since the early days of computational biology. Methods to avoid this problem can be classified into three approaches:

### Hard masking

The first approach is to identify low-complexity regions by some means, and then replace each letter in these regions with a dummy letter, typically X for proteins and N for DNA. During alignment, the dummy letter receives a negative match score when aligned to anything. For example, in the NCBI blosum matrices, X receives a score of −1. This prevents low-complexity regions from getting high alignment scores.

This approach obviously depends on the means of identifying low-complexity regions. We recently showed that standard methods such as SegMasker and DustMasker are not perfect: they fail to mask some low-complexity sequences, which then produce strong (E-value 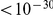), non-homologous alignments [1]. We also described a new masking method, tantan, which prevents non-homologous alignments much more reliably.

### Soft masking

The second approach is to indicate low-complexity regions with lowercase letters, instead of dummy letters. This leaves all options open: some alignment tools can treat lowercase identically to dummy letters. A popular method, however, is to exclude lowercase from the initial “seeding” phase of the alignment algorithm, but to treat lowercase identically to uppercase during the subsequent “extension” phase. (This only makes sense for alignment tools that use a seed-and-extend algorithm.) This method is used by blastz and lastz, which are employed to construct the widely-used UCSC genome alignments [2]–[4]. It is also used by the NCBI's blastn and megablast [5].

Unfortunately, excluding low-complexity regions from seeding but not extension fails to prevent spurious alignments [1], [6]. If we wish to thoroughly avoid non-homologous alignments, we must mask low-complexity regions at all stages of the homology search procedure.

It might be objected that masking at all stages of homology search will mutilate alignments of genuinely homologous sequences. It may break them into smaller alignments, and prevent alignment of mildly low-complexity regions whose homology is supported by surrounding high-complexity regions. We can avoid this mutilation as follows. After identifying homologous regions, re-align them with masking turned off, allowing the re-alignments to extend beyond the regions and merge nearby alignments. This re-alignment is naturally achieved by repeating the extend step of a seed-and-extend algorithm. We know of only two alignment tools that perform this careful variant of soft masking: fasta and last [1], [7].

### Compositional adjustment

The final approach is to adjust the alignment score and/or significance estimate, based on the letter frequencies. NCBI blastp (and tblastn [8]) can use either compositional scaling (a.k.a. composition-based statistics) [9], or compositional score matrix adjustment [10]. These methods aim not only to avoid spurious alignments, but also to discriminate homologs with non-standard letter frequencies in a statistically powerful manner. blastz and lastz adjust alignment scores based on the entropy of their nucleotides [11]. Among other tools, MSPcrunch filters alignments with biased letter frequencies [12], and hmmer3 uses a score correction for biased composition [13].

To the best of our knowledge, all methods in this category consider only zero-order letter frequencies, and not tandem repeats. Since tandem repeats often cause strong, non-homologous similarities [1], these methods do not suffice to avoid spurious alignments.

**Figure 1:**
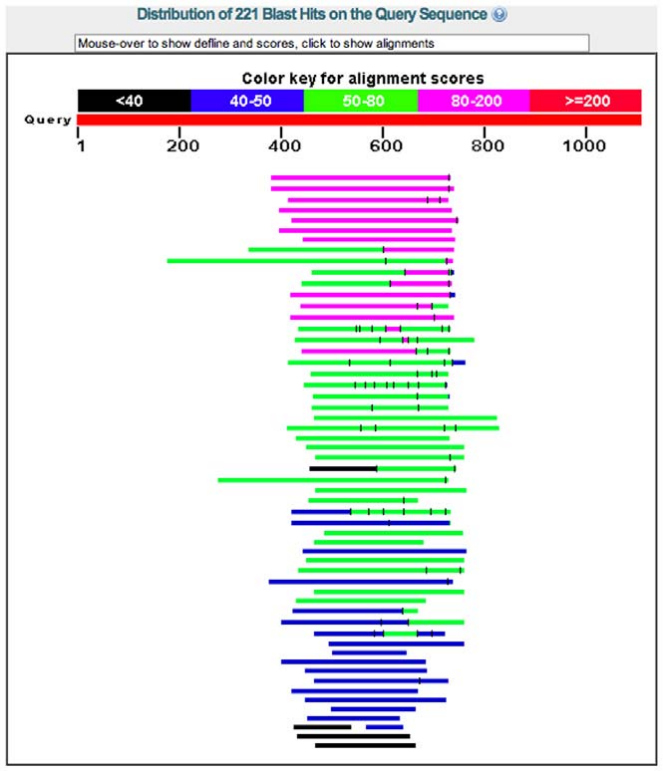
Spurious alignments found by BLAST. This is the output of a blastp search with a reversed protein (B6D5L7_PERAZ) against the nr database at NCBI.

For example, Figure 1 shows a blastp search with a *reversed* protein (B6D5L7_PERAZ) against the nr database, using “conditional compositional score matrix adjustment” (the default setting as of 2011-06-20). The search found 221 hits, including 21 with E-value less than 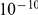. Since sequences do not evolve by reversal, these alignments are not homologies. These alignments are due to a period-28 tandem repeat in B6D5L7_PERAZ, which, after reversal, matches tandem repeats in other proteins (with period not necessarily 28, see Figure 2B of [1]).

**Figure 2:**
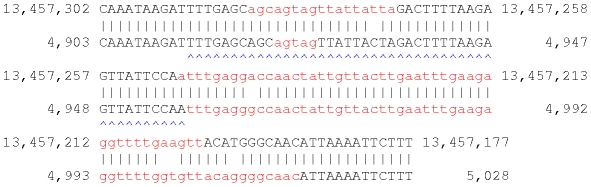
A NUMT in the X chromosome of *C. elegans*. This shows an alignment between the X chromosome (upper) and the mitochondrial chromosome (lower). Lowercase red letters were masked by tantan. The blue arrowheads indicate the first unit of an inexact tandem repeat.

### Summary

In summary, the only reliable way to avoid false homology predictions is to mask the sequences with tantan, and apply this masking at all stages of the homology search algorithm. (We have not tested all other methods that have ever been published, but we have tested several widely-used ones [1], [6].) Another way to state this is that tantan makes alignment E-values useful. For example, if we perform homology search using tantan and an E-value threshold of 10, the number of spurious alignments is likely to be around 10, and the strongest spurious alignment is unlikely to have an E-value much less than 1. Using a method other than tantan, the number of spurious alignments might exceed 1000, and the strongest one might have an E-value less than 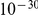 [1].

Since tantan typically masks less than 10% of letters, we do not expect it to greatly decrease the sensitivity of homology search [1]. We noticed, however, that it occasionally blocks some alignments that we suspect are true homologies. Here we present a new method, gentle masking, which rescues these blocked alignments. This does not change the procedure for identifying low-complexity regions, but rather it changes the way they are treated during homology search.

## Results

### A new method: gentle masking

The old method, “harsh” masking, assigns a negative score for a match between a lowercase letter and any other letter. In our previous publication on tantan, we used the lowest score in the scoring matrix. For DNA we usually use +1/−1 match/mismatch scores, so the score for a masked letter would be −1. For protein alignments with the blosum62 matrix, it would be −4.

With gentle masking, the score for matching two letters when either (or both) are lowercase is: 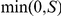, where 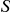 is the score when both letters are uppercase. We implemented this by enlarging the score matrix, to include separate entries for uppercase and lowercase letters (e.g. Table 1). This means that the alignment algorithm needs no change.

**Table 1:** Example of an enlarged score matrix for gentle masking.

	A	C	G	T	a	c	g	t
**A**	1	−1	−1	−1	0	−1	−1	−1
**C**	−1	1	−1	−1	−1	0	−1	−1
**G**	−1	−1	1	−1	−1	−1	0	−1
**T**	−1	−1	−1	1	−1	−1	−1	0
**a**	0	−1	−1	−1	0	−1	−1	−1
**c**	−1	0	−1	−1	−1	0	−1	−1
**g**	−1	−1	0	−1	−1	−1	0	−1
**t**	−1	−1	−1	0	−1	−1	−1	0

### Gentle masking improves the sensitivity of homology search

**NUMTs**: There have been many studies of NUMTs, which are copies of mitochondrial DNA in nuclear genomes [14]. A key step in NUMT identification is to find regions of the nuclear genome with homology to the mitochondrial genome, which is a standard homology search problem. (An additional step might be to distinguish transferred DNA from DNA that has been conserved since the common ancestor of eukaryotes and mitochondria: we do not attempt that here.)

We looked for NUMTs in several nuclear genomes, using either harsh masking or gentle masking of low-complexity regions identified by tantan. The difference is not great, but we found a few extra NUMTs using gentle masking (Table 2). In particular, we found the single previously-reported NUMT in *C. elegans* [14] only when we used gentle masking.

**Table 2:** NUMTs found with gentle or harsh masking.

Genome	NUMTs found with gentle masking	NUMTs found with gentle masking, but not found with harsh masking	NUMTs found with harsh masking, but not found with gentle masking
*C. briggsae*	4	0	0
*C. elegans*	1	1	0
*D. melanogaster*	6	0	0
*H. sapiens*	914	12	0
*M. musculus*	189	4	0

The *C. elegans* NUMT is not trivial to find, because large parts of it are deemed low-complexity by tantan (Figure 2). The un-masked parts of this alignment are collectively strong enough to be statistically significant: the alignment score with gentle-masking applied is 45, for an E-value of 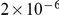.

The main reason tantan masks this NUMT is that it contains a period-45 tandem repeat. Such longish period repeats can indeed cause spurious alignments [1]. An accidental property of tantan is that it tends not to mask the left-most repeat unit (blue arrowheads). Although this is awkwardly asymmetric, it allows more sensitive homology search, as this example shows.

**Metagenomic DNA reads:** There is great interest in analyzing collections of DNA sequences from various environments, such as the human gut or seawater. In these experiments the sequencing instrument generates many short DNA reads, which must then be interpreted. One standard analysis is to align the reads to a catalog of microbial genome sequences, which may indicate the taxonomic groups that many of the reads come from. This is also a homology search problem.

**Figure 3:**
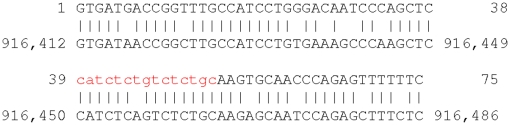
A metagenomic DNA read aligned to a bacterial genome. The upper sequence is the DNA read “1_lane2_104963”; the lower sequence is from the genome “A1-86”. Lowercase red letters were masked by tantan.

We aligned 1 million 75 bp Illumina reads to a catalog of 194 microbial genomes, using data conveniently provided from a previous study [15], [16]. In this test we used an E-value threshold of around 0.01 per read, which means that we expect about 10,000 spurious alignments. Using harsh masking, we matched 543,115 reads to at least one genome. Using gentle masking, we matched *all* of these same reads, plus an additional 703 reads. This is a very small increase in sensitivity, but an increase nonetheless. Only 6% of the reads contain tantan low-complexity regions, so the difference was bound to be small. Figure 3 shows an example that was found with gentle masking but not with harsh masking. As above, the un-masked parts of this alignment are collectively significant: the gentle-masked alignment score is 37, for an E-value of 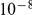.

As a negative control, we reversed the reads (without complementing them) and then aligned them to the genomes. With harsh masking 7,907 were aligned, and with gentle masking 7,910 were aligned. This suggests that gentle masking decreases the specificity of the search by less than it increases the sensitivity.

**Protein-coding homology**: It is useful to identify segments of a genome that are homologous to protein-coding DNA. Such segments are usually either protein-coding exons, or pseudogene fragments. One reason this is useful is to explain evolutionarily-conserved segments of the genome. Searches for conserved non-protein-coding segments tend to hit recent pseudogenes, because they have indeed been conserved for much of their evolutionary history. This is not always obvious, because comprehensive pseudogene annotation is lacking.

We identified segments of the human genome with protein-coding homology, by finding local alignments between the genome and known proteins in the UniRef90 database. We used “three-frame alignment”, which translates the DNA in all reading frames and aligns the amino acids allowing frame-shifts [17]. (We used both DNA strands, so six frames in total.) With gentle masking of low-complexity regions, we identified 225,002 genomic segments. With harsh masking, we failed to identify 6,422 of these (no overlap), and found only 2 segments not found with gentle masking. Thus, gentle masking slightly increased the sensitivity.

**Figure 4:**
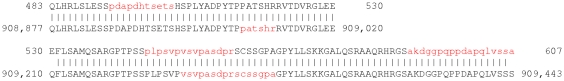
Alignments between a protein and the human genome. This shows two local alignments between a protein (Q494U1, upper sequence) and human chromosome 1 (lower sequence). Lowercase red letters were masked by tantan. The upper alignment was found with gentle masking, but not with harsh masking.

As an example, the upper alignment in Figure 4 was found with gentle masking, but not with harsh masking. This alignment corresponds to an exon of the known gene *PLEKHN1*. The lower alignment in Figure 4 shows the next downstream exon: this provides independent evidence that the upper alignment is not a spurious match.

It might be argued that our harsh masking is a straw man, because we used a mask score of −6 (the lowest score in blosum80), whereas, for example, blast uses a score of −1 for X residues. To address this concern, we repeated the search after replacing masked letters with X, with score −1. To make a fair comparison, we also repeated our gentle masking search without the final step of realignment without masking. In this case, with gentle masking we found 227,018 segments (more than before, because the final realignment step merges some segments). With X masking, we failed to find 1,640 of these, and found 43 segments not found with gentle masking. So gentle masking still improves the sensitivity.

### Gentle masking does not harm the specificity of homology search

It is impossible to prove that any method will always suppress spurious alignments: the best we can do is to test it on a variety of datasets. In order to provide as much support for gentle masking as we obtained previously for harsh masking, we repeated exactly the same tests as [1]. The short story is that the test results with gentle masking are almost identical to those with harsh masking, which means that gentle masking suppresses spurious alignments just as well.

In each of these tests, we look for local alignments between a set of reversed sequences and a set of non-reversed sequences. Since sequences do not evolve by reversal, there are no true homologies. With insufficient low-complexity masking, however, we observe strong alignments with significant E-values (Figure 1).

**Figure 5:**
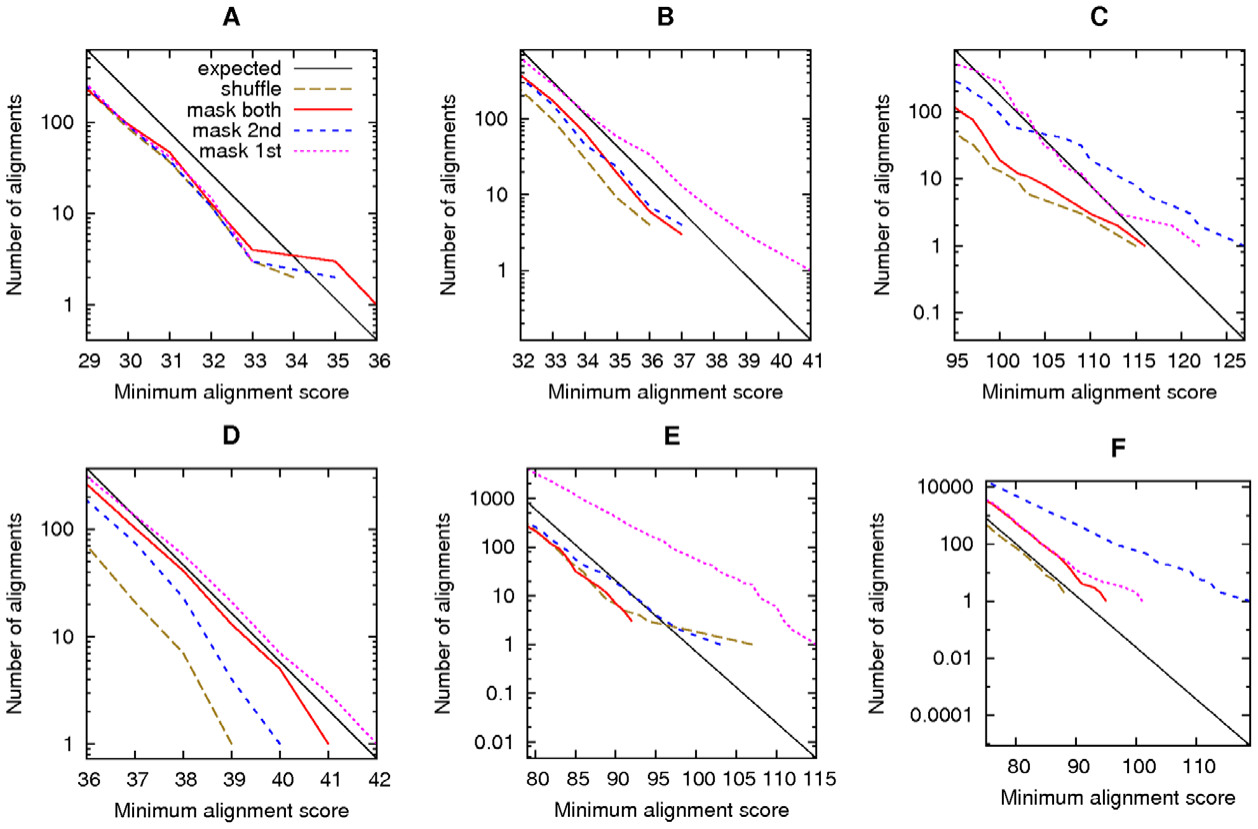
Alignments of reversed sequences, with gentle masking. This shows alignments between: (A) the *C. elegans* genome and the reversed *P. pacificus* genome; (B) the *A. thaliana* genome and the reversed *P. patens* genome; (C) vertebrate proteins and reversed plant proteins; (D) the human genome and the reversed opossum genome; (E) the *P. falciparum* genome and the reversed *D. discoideum* genome; (F) the *P. falciparum* genome and the reversed human genome. The colors indicate alignments after: masking both sets of sequences (solid red); masking the first-named set only (dotted magenta); masking the second-named set only (dashed blue); shuffling the letters in each set (dashed brown). The black lines indicate the expected number of alignments for random sequences.

In the first test, we compared the *C. elegans* genome to the reversed *P. pacificus* genome, gentle masking both with tantan (Figure 5A). The number of alignments (red line) agrees closely with the number obtained after shuffling the genomes (brown line) and the number expected from E-value calculations (black line). The number of alignments found after shuffling is less than theoretically expected because we used a heuristic search tool (last) which misses some alignments. In any case, spurious alignments were thoroughly suppressed.

We obtained similar results when comparing five other pairs of DNA or protein datasets (Figure 5B–F). The results are not “perfect”: for instance, in Figure 5D we clearly find more and stronger alignments in the reversed comparison (red line) than the shuffled comparison (brown line). Moreover, masking only one set of sequences in each pair (blue and purple lines) was sometimes less effective. In all cases, however, the results with gentle masking are extremely similar to our previous results with harsh masking (Figure 5 in [1]).

**Figure 6 pone-0028819-g006:**
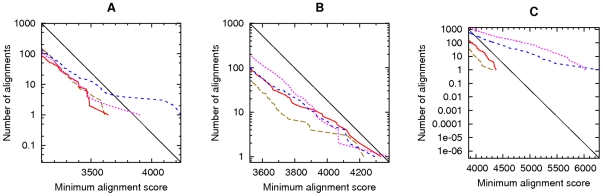
Alignments of reversed sequences, using the HOXD70 scoring scheme. Alignments between: (A) the *C. elegans* genome and the reversed *P. pacificus* genome; (B) the *A. thaliana* genome and the reversed *P. patens* genome; (C) the human genome and the reversed opossum genome. The colors indicate alignments after: masking both sets of sequences (solid red); masking the first-named set only (dotted magenta); masking the second-named set only (dashed blue); shuffling the letters in each set (dashed brown). The black lines indicate the expected number of alignments for random sequences.

The DNA alignments of Figure 5 all used the same scoring scheme: match = 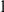, mismatch = 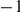, gap = 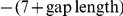. We repeated three of these tests using a different scoring scheme: the hoxd70 score matrix with a gap score of 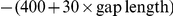 [11]. Again, we did not observe excessive spurious alignments after masking both sets of sequences (Figure 6, red lines). These results with gentle masking are also highly similar to our earlier results with harsh masking (Figure S7 in [1]).

**Figure 7:**
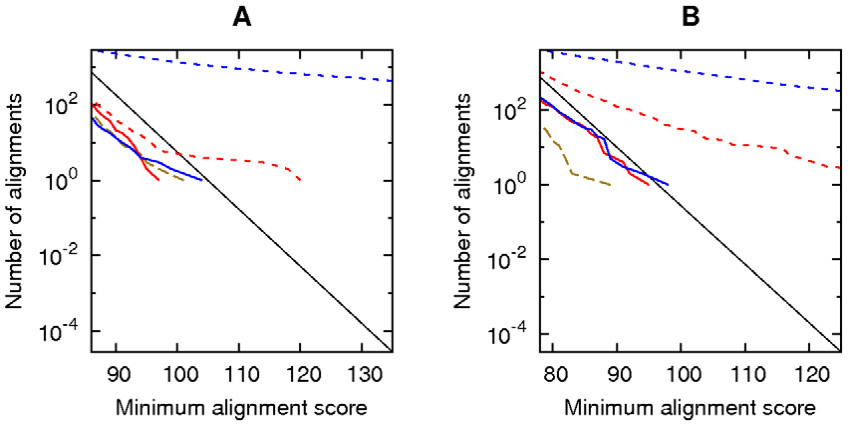
Alignments between DNA sequences and reversed protein sequences, with gentle masking. This shows alignments between: (A) the *C. elegans* genome and reversed plant proteins; (B) the *P. falciparum* genome and reversed vertebrate proteins. The colors indicate alignments after: masking the proteins, and the DNA at the protein level (solid red); masking the proteins, and the DNA at the DNA level (solid blue); masking the proteins only (dashed red); masking the DNA only, at the DNA level (dashed blue); shuffling the letters in each set (dashed brown). The black lines indicate the expected number of alignments for random sequences.

Finally, we compared DNA sequences to reversed protein sequences (Figure 7). In order to exactly mimic the test in our previous publication, we did not allow frameshifts. Once again, the results with gentle masking are extremely similar to those with harsh masking (Figure 7 in [1]).

## Discussion

### Why not use a mask score of 0?

**Figure 8:**
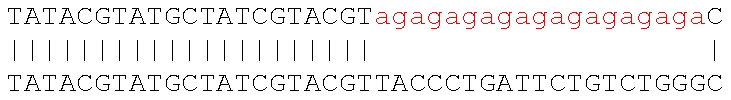
Alignment problem using a mask score of 0. This kind of nonsensical alignment may occur if masked letters (lowercase red) always receive a score of 0.

An alternative to gentle masking is to assign a score of zero for a match between a masked letter and any other letter. This idea has several problems, however: it leads to over-extended alignments such as that in Figure 8, and it makes blast-like algorithms slow because they would explore alignment extensions across the whole length of every masked region that they encounter. It also complicates the final step of realignment without masking, because optimal unmasked alignments might be multiple fragments of an optimal masked alignment. Our gentle (and harsh) masking method avoids this problem by guaranteeing that the masked score cannot exceed the unmasked score.

### Masking versus modeling

A more sophisticated way to avoid false homology predictions would be to use probabilistic models. The standard approach to sequence alignment, using a score matrix, can be interpreted as comparing a model of related sequences to a model of independent sequences [18]. It might be possible to incorporate low-complexity regions into both of these models. In fact, tantan is based on a probabilistic model of sequences with low-complexity regions [1]. Thus, it might be possible to construct a principled and extremely accurate homology search method by combining the tantan model with the alignment models.

The main advantage of gentle masking is that it actually exists now. (last enlarges the score matrix as in Figure 1.) The modeling approach seems to us not entirely easy to implement. It would surely complicate the alignment algorithm, perhaps making it slow, and making it difficult to retrofit into existing methods like blast. This would hamper its adoption by the bioinformatics community.

### Profile-based homology search

Profile-based methods are often more powerful than pairwise sequence comparison at finding remote protein homologs [9], [13], [19]. For methods like psi-blast that use a position specific scoring matrix (PSSM), gentle masking generalizes in an obvious way: if 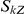 is the PSSM score for (uppercase) letter 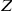 at position 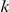, then 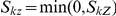. On the other hand, it is not obvious whether or how one should identify low-complexity tracts in a PSSM. For methods like hmmer that use explicit probabilistic models, it seems more logical to incorporate a model of low-complexity regions, as mentioned in the preceding subsection.

Pairwise sequence comparison (without profiles) remains frequently useful, as illustrated by the three examples in this study. Moreover, pairwise sequence comparison is needed for gathering homologs to construct profiles in the first place. In this homolog-gathering step, it is often particularly important to avoid contamination by non-homologous sequences: our masking approach should be very useful here.

### Interspersed repeats are not low complexity

Interspersed repeats and low-complexity sequences are often lumped together as “repeats”, so we must constantly point out the differences between them. Interspersed repeats (such as LINEs and SINEs) do not cause false homology predictions, because, for example, every LINE-1 element is genuinely homologous to every other LINE-1 element. They may cause other problems, such as making sequence comparison algorithms run too slowly and produce too much output. So it can be useful to lowercase-mask both low-complexity regions (using tantan) and interspersed repeats (several tools exist). Gentle masking would then operate on both types of repeat, which seems harmless. (In fact, we did this in our protein-coding homology search.)

### Masking and orthology search

Low-complexity masking is important for accurate homology search, but its application to orthology search is less clear. Prominent examples of orthology search include: comparing two whole genomes, and aligning human DNA reads to a reference human genome. In these cases, we usually wish to avoid paralogous alignments. The danger is that we might mask an ortholog but not a paralog, and thus increase the rate of paralog alignments. This seems especially likely with short sequences, where any masking is more likely to cover the whole ortholog. On the other hand, if we have DNA reads with contaminants (e.g. bacterial), there is a risk of spurious low-complexity matches between the contaminants and the genome. We speculate that it might be useful to apply low-complexity filtering as a final step, after identifying orthologs.

### Homology search versus alignment

Homology search has two somewhat different aspects: 1) finding homologous regions, and 2) aligning homologous letters within those regions. This study addresses only the first aspect. There have been several studies on the accuracy of letter alignment (e.g. [6], [20]), but to our knowledge none have examined the effect of low-complexity tracts. Such tracts are likely to have a significant effect: for example, they are likely to exacerbate over-extension of local alignments [21]. Research into the effect of low-complexity tracts on letter alignment would be useful.

### Conclusions

Gentle masking is an extremely simple but useful way to treat lowercase-masked low-complexity tracts during sequence alignment. In tests with reversed sequences, gentle masking with tantan suppressed spurious alignments in a practically identical manner to harsh masking with tantan. On the other hand, in three tests using real (non-reversed) sequences, gentle masking resulted in slightly but noticeably more alignments than harsh masking. Since both methods suppress spurious alignments equally well, we infer that these extra alignments are largely true homologies. In support of this conclusion, some of the extra alignments have highly significant E-values (which are never observed for reversed sequences), and some of the putative protein-coding homologs are supported by neighbouring exons.

## Materials and Methods

### Tests of specificity

To obtain the results shown in Figures 5–7, we used the same materials and methods as in our previous publication [1].

### Tests of sensitivity

**All:** In all of these tests, we masked both sets of sequences (query and reference) using tantan version 4 [1]. We found alignments using last: version 163 for harsh masking, and version 164 in all other cases [22]. E-values were calculated with lastex [23].

In an abundance of caution, we made sure that the DNA strands were treated symmetrically, despite tantan's directional asymmetry. We first compared forward strands only, using lastal option -s1. We then reverse-complemented the original (un-tantan'd) query sequences, ran tantan on these, and fed them to lastal using option -s1 again.

This cautious treatment of strands is the reason why our results with the metagenome data are not identical to the results we obtained earlier [24].

**NUMTs:** We downloaded these genomes from the UCSC genome database: cb3, ce6, dm3, hg19, mm9 [25], [26]. The mitochondrial genomes are circular, but are represented as linearized sequences. Therefore we doubled these sequences, in order to find alignments that cross the break.

We set the alignment score threshold to the minimum score with E-value
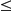
0.01. For example, the score threshold for *C. elegans* was 36. (E-values were calculated before doubling the mtDNA.)

We obtained a count of NUMTs as follows. We recorded the segment of the nuclear genome covered by each alignment, discarding the mitochondrial coordinates and all strand information. We then merged overlapping and touching segments. Finally, NUMTs found by one method but not the other are defined to be segments found by one method that have no overlap with segments found by the other.

**Metagenomic DNA reads:** One of the genomes that we downloaded (Acidaminococcus_D21) has other genomes spuriously appended to it, so we used only the first 2,238,973 bases of this sequence.

We ran lastal with options -d20 -e25. This means that we used a score threshold of 25, for a E-value of about 0.0188 per read.

In the test with reversed reads, masking was done before reversal.

**Protein-coding homology:** We obtained UniRef90 from UniProt release 2011_05 [27], and hg19 from the UCSC genome database [25], [26].

The proteins were masked using tantan options -p -r0.02, as recommended for DNA-versus-protein alignment [1]. The DNA was masked using option -c, which preserves the lowercase masking done by UCSC. We did this because a few proteins match interspersed repeats (due to exaptation), and we wished to avoid these numerous alignments.

We aligned the DNA and the proteins using lastal with options -pBLOSUM80 -F15 -e137. The score threshold of 137 corresponds to an E-value of about 0.01.

**Figure 9:**
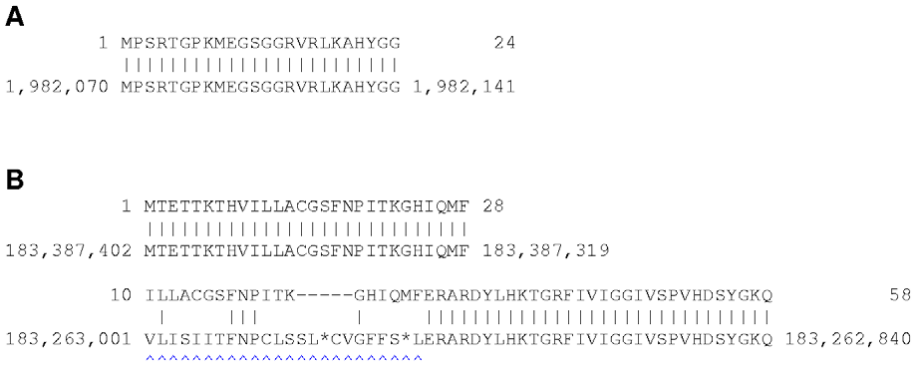
Alignments using BLOSUM80 or BLOSUM62. (A) An alignment that is significant when scored with blosum80 but not blosum62. (B) Two local alignments between a protein (Q9BZQ4, upper sequence) and human chromosome 1 (lower sequence). The blue arrowheads indicate spurious over-extension of the second alignment. This over-extension occurs when the blosum62 matrix is used for alignment, but not when blosum80 is used. (Actually, it is conceivable that the extension correctly indicates homology: the genomic segment marked by blue arrowheads could be paralogous to the genomic segment in the first alignment.)

We used blosum80 instead of the more standard blosum62 for two reasons. First, blosum80 is more powerful at discriminating short, strong homologies from chance matches (e.g. Figure 9A) [18]. Second, blosum62 is more prone to over-extending alignments (e.g. Figure 9B) [21].

We counted protein-coding segments in the same way that we counted NUMT segments, with one difference: this time, we treated segments on opposite strands of a chromosome as distinct.
